# Meeting Reports

**DOI:** 10.1155/S1463924679000304

**Published:** 1979


### Meeting Reports

### Laboratory Management and Automation

The 8th Technicon International Congress, was held from the 12th-14th December, at the Wembley Conference Centre, London, UK. It is over seven years since such an event was last held in the United Kingdom and over the three days of the congress a wide range of analytical topics covering both industrial and clinical chemistry were covered in the various sessions. In addition the delegates were able to browse around the complete exhibits of Technicon products and to discuss various aspects of their work with the Technicon staff in attendance. As one has come to expect from such conferences it was extremely well organised and Technicon should be congratulated on this aspect. The technical papers were informative if sometimes lacking in originality. The papers did however prompt interesting discussions and the panel sessions were a worthwhile and stimulating adjunct to the meeting. The complete presentations will be published shortly. In addition it is hoped that some of the more original works will be published in The Journal of Automatic Chemistry. It is therefore not the aim of this report to cover the entire meeting programme, rather to deal with those lectures that appealed to the various correspondents or when points were made of more general interest.

The balance of papers between the clinical and industrial applications increasingly becomes more equable which whilst it cannot reflect the revenue from instrument sales shows how important the management of Technicon considers the industrial market. The fully automated HPLC system and the more versatile Infra-Analyzer systems shown will surely have a large impact on the introduction of automation in many new areas at present reliant on inadequate manual analyses. Data Management and computer applications were considered in some detail if only in a clinical context. One of the main objections to computers is that they are shrouded by jargon used by computer specialists to protect themselves. This often has a self defeating role and people resist becoming involved in new technology because of it. It is strange therefore that clinical chemists have compounded the problem more by considering the application of computers to clinical laboratories quite in isolation from other analytical chemists. Handling data from an AutoAnalyzer, be it dealing with clinical or industrial analysis, is a similar problem, and many industries deal with large amounts of data and data banks. There is surely much to be gained from a cross fertilization of ideas and little by further hiding behind another level of jargon.

The symposium sessions were opened with a welcoming address by the Managing Director of Technicon, Mr. Tony Scobie. He then introduced Mrs. Louise Evans who presented the 1978 Technicon Award to Professor J. R. Daly, University of Manchester, UK. Mr. Scobie then introduced Mr. Jack Whitehead, Chairman, The Chief Executive of Technicon who spoke for a few minutes on the Company's future programme.

The plenary paper 'Food Analysis in the Wind of Change' was then delivered in an interesting and informative manner by Dr. Magnus Pike. He put forward fairly innovative and informed views which will no doubt cause much food for thought by quality control managers and the like.

The main congress proceedings were then organised into parallel sessions around various subject themes. The titles of these sessions were: Laboratory Data Management, Automated Investigation in blood banking, Economics of Analysis in Commodity Trading and Food Processing, Current Trends in Clinical Chemistry, Automation in water monitoring and sewage control, Developments in Automated Haematology, New Windows in Clinical Chemistry, Automation and Application of Immunoassay, Automated Analysis for intensive care and Pharmaceutical quality control. These lecture sessions were further supplemented by evening workshops which were also very well attended.

Dr. R. Scholtis, the Director of a large medical laboratory in the Netherlands, adopted a humorous, but nevertheless informative and very useful approach to describing the introduction of computers in his laboratory. He traced the development from the planning stages in 1965 through to the implementation in 1971, the addition of online automation, and the system up-grade in 1976. Dr. Scholtis concluded with a number of general observations that will be of considerable interest to anyone contemplating following a similar path.

(1) Hardware is relatively cheap — programming is very expensive.
(2) Once you start you cannot go back — the work load increases so much that reversion to manual techniques becomes physically impossible.
(3) Don't play with "home-made" computing systems. They are not cost-effective.
(4) Go to a good and not a big company.
(5) Find someone to advise you who has experience in computers.

Mr. P. Tong, President of Technicon T & T Corporation, said it was impossible to build a universal computer system that will meet the demands of all laboratories. He described the family of products Technicon were designing to meet the requirements of most hospitals. Only industry standard hardware was used. Software was written in an interpretative language — MUMPS. It includes extensive personnel management software, for example worklists could be generated on demand. Positive sample identification was incorporated and the use of Optical Character recognition was planned.

Professor T. P. Whitehead made two telling points, he considered the output capabilities of visual display units and graph plotters indispensible and that quality control could be greatly improved using the range of data reduction tools available. Dr. B. Leijnse failed to answer the question of whether or not we needed the computer. He pointed out however that the clerical work had increased in the hospital situation and that once committed to a computer situation it is impossible to consider coping without it.

There were several interesting papers relating to the design and applications of the technique of reflectance infra red measurements embodied in the InfraAlyzer range of instruments.

Mr. C. Maddix, Director of the Technical/InfraAlyzer project, described the InfraAlyzer 400 infra red reflectance instrument. He stated that the qualities needed by an instrument for this technique were speed, accuracy and precision, flexibility, simplicity (particularly for operation by plant workers) and reliability. He then described how the InfraAlyzer 400 fulfilled all these criteria and also how the use of microprocessors had aided this development. They have provided very sophisticated calculating and computing functions and complete fault diagnosis functions.

Mr. B. Bruinsma of the Food Science Dept., Washington University, then described the use of an infra red reflectance instrument for the analysis of grain. For years his department had been analyzing 20,000 samples a year for protein and of these 2,000 a year were checked by Kjeldahl. His instrument had been calibrated for protein and protein-plus-moisture for twelve commodities. The range of analytes had also been extended to cover lysine determinations and as a hardness gauge for wheat and cereal crops.

Dr. H. Swan of the Nottingham University School of Agriculture described his experience in the use of the InfraAlyzer in the analysis of cattle feed. Two factors were of direct interest, the energy available to the animal and the protein (ultimately amino acid) supply. Since the feed bill for the UK was of the order of £1,000M, a saving of 10% was very significant and whilst Dr. Swan felt that the technique was in its infancy it had considerable potential in this field. Applications of the Infra-

Alyzer in the tobacco and dairy industries were than discussed by messrs Long and Weaver respectively. Both felt that recent improvements and the isolation of more selective wavelength regions pertinent to their own products offered greater potential in the future.

Dr. P. Wilding, Director of Programs/Reagents & Biologicals, Technicon, USA, discussed the considerable effort in recent years that had been put into reducing the reagent consumption of automatic analysers. The obvious advantage was a direct savings in reagent cost, less obvious but also important was the indirect cost saving in terms of the space required to accommodate large volumes of prepared reagent solutions. Perhaps the ultimate in space and reagent economy was the bound hexokinase coil which lasted for 4 weeks in routine use.

Dr. Elizabeth Shaw, of St. Bartholomew's Hospital, said that although radioimmunoassay techniques were a considerable advance in laboratory analysis they had a number of practical disadvantages. She described the development of an immuno-assay, using fluorescein as a marker that overcame these problems. This is easily automated and Dr. Shaw described the application of this new technique to the assay of the antibiotic gentamicin.

Dr. D. Burns, Director R & D, Technicon Industrial Systems, USA, described the new Technicon automated HPLC system in the session devoted to liquid chromatography. But a more general application was presented by Professor R. Frei of the Department of Analytical Chemistry, Free University, Amsterdam, who said that three types of derivatisation could be used in HPLC: pre-column, in situ, or post column derivatisation. He discussed examples of post column detection systems working on both segmented and unsegmented continuous flow principles with particular emphasis on the practical aspects. The technique had two advantages:
(1) the formation of artefacts does not usually interfere and
(2) different detection principles can be employed simultaneously.
Among disadvantages were:
(1) the diluting solvent could impose limitations on the reaction medium
(2) the reaction must be very rapid.

*J. Holme*

### Automation in Atomic Spectroscopy

A meeting on this subject organised jointly by the Automatic Methods and Atomic Spectroscopy Groups of the Analytical Division of the Chemical Society was held in the Geological Society Lecture Theatre at Burlington House, Piccadilly, London on 7 December 1978.

The meeting opened with a paper entitled 'Computer assisted geochemical analysis with an atomic absorption spectrophotometer' in which Dr. A. N. Baxter of the City of London Polytechnic described the analytical techniques used in his work. The second paper was presented by Dr. J. Warren, entitled 'The determination of trace metals in food using an automated digestion and extraction system, followed by plasma emission spectroscopy'. A system, developed and built at The Laboratory of the Government Chemist, London, was described. The system automatically digests samples at the rate of three per hour, neutralises the digest by the controlled addition of anhydrous ammonia, extracts the metals as their chelates into organic solvent and separates and collects the resulting organic extract. Dr. Warren went on to describe subsequent analysis of the extract with an ARL inductively-coupled plasma optical emission spectrometer capable of simultaneous 20-channel measurement. The spectrometer was linked to the Laboratory Xerox Data Services — 530 computer for subsequent data handling and reporting. Finally Dr. M. D. Sylvester of Plasmatherm Inc., described a system used by him in Canada for geochemical prospecting. A sampling system carried in a light aircraft flown at a low altitude was used to trap particulate matter in the atmosphere on a reel of adhesive tape. Subsequently this tape was analysed in an instrument where a laser beam vapourised the sample from the surface of the tape and into a plasma-emission spectrometer.

*D. G. Porter*

### Microprocessors and the Chemist

A symposium with the above title was held by the Chemical Society at Atomic Energy Research Establishment (AERE) Harwell on 18 January, 1979. The Symposium was well attended with a well-balanced audience from industry, government bodies and education; which indicated the current wide-spread interest in the subject matter. The morning's proceedings were opened by Dr. T. Pearce of AERE Harwell, the local organiser, who described the aim of the Symposium as being two-fold. Firstly, to show the chemists the 'current state of the art' and to indicate how micro-processors can assist the chemist, and secondly to gauge the measure of interest in forming a subject group of microprocessors within the Chemical Society. The morning lectures were con-cerned with the hardware aspect of microprocessors and the novel home computers, while the afternoon lectures dealt with particular applications involving microprocessors. The first paper entitled 'Introduction to microprocessor systems', given by Dr. D. Newton, also of AERE Harwell, was a lucid and brief review of microprocessors which formed an admirable back-ground for the afternoon lectures. The second of the morning lectures was given by Mr. M. Whitbread of the National Comput-ing Centre, Manchester, on 'Personal computers'. He described both systems currently available together with the interfacing required between home computers and data sampling devices.

The afternoon was introduced by Dr. J. Barratt, whose initiative had launched the meeting. The lectures had been chosen to give a broad spectrum of applications in order to show the full potential of microprocessors in chemistry. Dr. D. R. Deane from the Petrochemical Division of ICI gave a paper entitled 'Micro-processors and scientific research' which had the underlying theme that chemists should purchase ready-made computers rather than trying to build them from kits and that micro-processor controlled instruments should be bought wherever possible. Although he was in favour of applying home computers to 'home-made' scientific equipment, the use of microprocessors in holding laboratory records and in any administration was strongly recommended. In a lecture on 'Microprocessors and computer-aided learning techniques', Professor P. Ayscough from the University of Leeds described the future use of micro-processors in the role of assisting teachers and concluded that their advent in the classroom would hopefully result in more individual tuition. An account of 'Microprocessors and analysis' was given by Dr. D. Betteridge from University College, Swansea, who has for the last 6 years, been involved in applying microprocessors to instrumentation. He agreed with the view of Dr. Deane that a system involving a microprocessor should be developed from scratch rather than adapting traditional equip-ment and described two such projects supervised by himself, an automatic titrator which is commercially available and a viscometer employing flow injection analysis, which is still in the research stage.

The final two papers; 'Microprocessors and process control' given by Dr. P. King of Warren Spring Laboratories, and 'Micro-processors and computer-aided process plant design' by Dr. P. Winter of the National Computer-aided Design Laboratory, Cambridge, were respectively concerned with the application of microcomputers to the control of large scale industrial plants and their design. However, it was concluded that although micro-processors will, in future, be capable of controlling industrial plants, home computers will not be sufficient for either application.

*T. Lenea Goad*

